# Cases from My Note-book

**Published:** 1883-11

**Authors:** Julius Schmitt

**Affiliations:** Rochester, N. Y.


### CASES FROM MY NOTE-BOOK.

JULIUS SCHMITT, M. D., ROCHESTER, N. Y.

2d October, 1880.—Mr. G. S., 40 years old, farmer, hard-working, robust man of dark complexion and jovial temperament, complains of stitches into right ear, going into the very brain, as he expresses it. They commenced several days ago, at 9 A. M., while he was working in the field, and the left ear was affected at first; this is now free from pain.

The stitches are worse at night.

There are small pustules on left wrist, which, when opened, discharge a milky fluid. Before this he had panaritia of left thumb and left middle finger.

Since he has had the typhoid fever, in 1873, he has been more sensitive to changes in the weather, and he also states that for the last few years *bee-stings will bother him more than they used to do.* ℞ Sepia$^{2c}$, 6 powders, one every second day.

18th of October, 1880.—Pains are still in the right ear, but less in severity and not so steady. Last week he noticed a furuncular swelling on left upper leg, which, however, disappeared without breaking.

Soreness of right outer canthus, sclerotica somewhat injected. Sensation as if something were before the right eye, when looking into the bright light. ℞ Sepia$^{2c}$, one dose.

Was cured within the next four weeks. Seeing him a year ago, I inquired about his sensitiveness against bee-stings. The answer was, "Oh! I can go now to any bee-hive, and am not afraid of their stings any more."

This case is brought to your notice on account of the symptom, *more sensitive to bee-stings than usual*, which, in this case, was the key-note for Sepia. We find in *Allen's Cyclopœdia*, under symptom 2009, the following: "After being stung by a bee, redness and itching red rash over the whole body, inflamed eyes, drops of sweat on the face, all coming on in a few minutes."

These same symptoms are incorporated in the provings of Sepia in Hahnemann's *Chronic Diseases*, and were looked upon by me with a little skepticism, bearing in mind that Hahnemann was yet unacquainted with the provings of Apium virus. The more rejoiced I was, therefore, to find that even here, when there might have been a reasonable doubt, the observations of this greatest of observers were correct.

CASE II.—24th February, 1882.—Mrs. Carry B., 24 years old, dark hair, spare habit, has been sick for eleven months under "scientific" treatment. Trouble commenced third day after delivery of her first and only child, which was helped into this world *by means of the forceps.* Status presens: Discharge of pus from vagina and rectum—from the former continually, from the latter only with movements of bowels, which are otherwise regular. Paroxysmal pains in abdomen, commencing in right ovarian region, going across to other side, sometimes spreading into back and legs. There is a steady pain in right ovary; she cannot lie on that side, and finds the most comfort when lying on back and *very flat, must even have the pillow removed from under head.* Micturition scanty; painful at times; used to be very painful. Urine is red and leaves a purulent sediment at the bottom of vessel.

She sleeps fairly and wakes up a good many times. *Sleep does not do her much good; awakens tired, and limbs pain as if pounded.* Her feet are always cold and dry at night.

She feels chilly, and likes a comfortable, warm room. Tongue coated white in middle, borders red and shining; papillæ raised at apex, and tongue, when protruded, catches a little behind the teeth.

She is very low spirited, inclined to crying spells, and hopeless. ℞ Arnica$^{cm}$ (Swan), one dose and placebos.

28th February, 1882.—For three days she has had most fearful pains, worse than she has for a long time, but last night she slept better than she has done since she was sick.

Discharge from vagina is less. She has not had any paroxysms of pain to-day; feels better after sleep; more refreshed. ℞ Placebos.

25th of March, 1882.—She has been steadily improving without any more medicine. Discharge from vagina and rectum has ceased entirely; no pains; appetite is good; bowels and water regular.

She complains to-day of redness of both eyes, cannot stand the light. Headache almost every day, commencing in occiput, spreading to forehead; tongue coated white in middle, borders red; papillæ of apex prominent. Hungry, gone feeling between ten and eleven A. M. ℞ Sulfur$^{cm}$.

11th of September, 1883.—Got entirely well and has not been sick since. Received no more medicine.

